# Annotations

**Published:** 1904-08-06

**Authors:** 


### Annotations.

### What the People Sleep On.

Under this title Mr. Peter Fyfe, the sanitary inspector of Glasgow, related to the Congress of the Sanitary Institute, recently held in that city, the results of an investigation he had conducted into the nature of the materials used as bedding by the poorer classes. Of 3,163 beds examined 2,471 were found to consist of a material known as "common flock." This substance, it appears, is manufactured largely from rags of cast-off clothing obtained from all ranks of the population. These, without any attempt at cleansing or disinfection, are converted by the aid of machinery into "flock." The condition of the raw material as examined at one of the factories visited was filthy in the extreme; and half a pound of the "flock" treated with a considerable quantity of distilled water gave a fluid which compared very badly with a similar volume of average Glasgow sewage. Of albuminoid ammonia the fluid gave 2·002 as compared with ·236 for the sewage, and whilst the chlorine yielded by the latter was 12·6 the figure for the bedding was 22·4. The total suspended solids in the crude sewage was 24·44 grs. per gallon, and in the rinsings from the "flock" 227·07 grs. per gallon. Any suggestion that the specimen examined was probably a particularly bad one was met by the fact that a new flock bed, a bolster, and two pillows obtained from one of the largest City furnishers yielded an even worse result. Bacteriological examinations conducted by Dr. R. M. Buchanan showed a condition shocking to contemplate, and supported Mr. Fyfe's conclusion that a bed filled with sewage ought to be preferred to the material on which 78 per cent. of the humbler Glasgow citizens are accustomed to sleep. The excuse offered by the manufacturers of this material is that commercial competition compels them to use it. If the Glasgow maker declines to do so, he finds he is cut out of his own market by bedding made in Yorkshire. Mr. Fyfe by dragging this revolting practice into the light of day has rendered the public good service, and if the legal powers of the local health authorities are not sufficient to deal with so flagrant an evil, the sooner they are enlarged the better.

### Instruction in Hygiene and Temperance.

The organisers of the petition to the educational authorities praying that instruction in temperance and hygiene may be included in the compulsory curriculum of elementary schools are certainly to be congratulated on the fact that they have secured the sympathies of nearly 15,000 registered medical practitioners. It is also a source of satisfaction that the influential deputation which supported the petition obtained so ready and sympathetic a hearing from Lord Londonderry, who has previously shown a keen interest in the physical welfare of the school population under his official care. There now remains the necessity to find the ways and means to translate these praiseworthy ideals into practice. And it would appear that upon this aspect of the subject there is some misapprehension and even conflict of opinion. From a question recently asked in the House of Commons it is evident that there are those who consider that the proper basis for such instruction is a knowledge of the elementary scientific facts of physiology and hygiene. Hence the suggestion that lectures on these subjects should be given to the scholars by medical practitioners. There are other indications that some of the supporters of the movement favour such a view, as, for example, when one of the members of the deputation urged that children should be taught "that alcohol, next to the microbe, is the commonest cause of disease, of poverty, and of crime." We have previously expressed our opinion as to the right method to be pursued in the education of children in temperate and healthy living, and this is in direct opposition to a suggestion that they should be introduced to the facts of physiology and pathology. Such facts will never touch the sympathies and interest of the youthful mind, and no stimulus to clean living will be provided by the statistics of disease of pauperism or of crime. Indeed, any such course is likely either to remain as a mere technical lesson of no practical effect or to produce morbid and unhealthy mental habits. What is wanted is not the provision of technical knowledge but the presentation of good health, moderation, and self-control as attractive virtues which rest on an ethical basis and from which a wilful departure is shameful and unworthy.

### "How to Help our Fellows."

Sir Oliver Lodge, it appears from an article in the *St. James's Gazette* of last week entitled as above, is of opinion that the entertainment and recreation of the people after their day's toil is too important a matter to be left wholly to private enterprise. He seems to intend to advocate the supply of places of recreation, at which food and drink could be obtained, and at which music and theatrical entertainments would be provided, as part of the ordinary duty of municipalities; and he indicates that too much pains need not be taken to render such places remunerative, nor even self-supporting. We are told explicitly that the entertainment department should be conducted independently of considerations of profit, which are said to be fatal to "art"; and, if the restaurant department were conducted on similar principles, we should at least approach to conditions which existed in an earlier civilisation, and the effects of which were not entirely satisfactory. *Panem et Circenses* were all very well while they lasted, but they did not last for ever, and, in the long run, they had to be paid for by somebody, and to be paid for at a ruinous price. Sir Oliver is quite right in attributing much misery to an unnecessary want of home comforts among the wage-earning classes; and nothing would be so certain to increase the standard of home comfort as the careful instruction of their girls in cookery and domestic economy, even at the cost of neglecting the piano and the elements of geology. A working-man whose wife was independent of preserved provisions and of advertised filth, and would give him an appetising meal at a cost within his means, would be half rescued from the publican and the tobacconist. If Sir Oliver will bend his energies in this direction, he will be likely to do more good than by the advocacy of municipal music-halls, and municipalities will be free to turn their attention to that great problem of better housing, which, if it were solved satisfactorily, would render music-halls superfluous.

